# The Guideline Language and Format Instrument (GLAFI): development process and international needs assessment survey

**DOI:** 10.1186/s13012-022-01219-2

**Published:** 2022-07-19

**Authors:** Samir Gupta, Rosalind Tang, Kadia Petricca, Ivan D. Florez, Monika Kastner

**Affiliations:** 1 Li Ka Shing Knowledge Institute of St. Michael’s Hospital, Unity Health, Toronto, ON Canada; 2 Division of Respirology, Department of Medicine, University of Toronto, Toronto, ON Canada; 3 Department of Geriatrics, Guy’s and St Thomas’ NHS Foundation Trust, London, UK; 4 King’s Health Partners, King’s College London, London, UK; 5 Centre for Global Child Health, The Hospital for Sick Children, Toronto, ON Canada; 6 Department of Pediatrics, University of Antioquia, Medellín, Colombia; 7 School of Rehabilitation Science, McMaster University, Hamilton, ON Canada; 8 Research and Innovation, North York General Hospital, Toronto, ON Canada; 9 University of Toronto, Toronto, ON Canada

**Keywords:** Clinical practice guidelines, Implementation science, Guideline implementation

## Abstract

**Background:**

Successful guideline implementation depends both on factors extrinsic to guidelines and their intrinsic features. In the Guideline Implementability for Decision Excellence Model (GUIDE-M), “communicating” content (language and format) is one of three core determinants of intrinsic implementability, but is seldom addressed. Our aims were to develop a tool that could be used by guideline developers to optimize language and format during development; identify gaps in this type of guidance in existing resources; and evaluate the perceived need for and usefulness of such a tool among guideline developers.

**Methods:**

Our mixed-methods design consisted of (1) content development (selection and organization of evidence-based constructs from the GUIDE-M into a prototype Guideline Language and Format Instrument (GLAFI), followed by face validation with guideline developers); (2) document analysis (duplicate) of seven existing guideline tools to measure coverage of GLAFI items and identify new items; and (3) an international survey of guideline developers (corresponding authors of recent Canadian Medical Association or Guidelines International Network database guidelines) to measure perceived importance of language and format, quality of existing resources, and usefulness of a language and format tool.

**Results:**

GLAFI items were organized into 4 language and 4 format subdomains. In face validation with guideline developers (17 clinicians, 1 methodologist), all agreed that the tool would improve guideline implementability and 93% indicated a desire for regular use. In the existing guideline tool document analysis, only 14/44 (31.8%) GLAFI items were operationalized in at least one tool. We received survey responses from 148/674 (22.0%) contacted guideline authors representing 45 organizations (9 countries). Language was rated as “extremely important” or “important” in determining uptake by 94% of respondents, and format by 84%. Correspondingly, 72% and 70% indicated that their organization would likely use such a tool.

**Conclusions:**

Optimal language and format are fundamental to guideline implementability but often overlooked. The GLAFI tool operationalizes evidence-based constructs, most of which are absent in existing guideline tools. Guideline developers perceive these concepts to be important and express a willingness to use such a tool. The GLAFI should be further tested and refined with guideline developers and its impact on end-users measured.

**Supplementary Information:**

The online version contains supplementary material available at 10.1186/s13012-022-01219-2.

Contributions to the literature

- Although research has identified three intrinsic features determining “implementability” of a guideline (developers, content creation, and content communication), communication has not been the focus of existing guideline development tools
- We built the Guideline Language and Format Instrument (GLAFI) by extracting actionable constructs from an implementability model (GUIDE-M), refined it based on user feedback and existing tool analysis, and demonstrated a lack of comparable guidance in existing tools along with high perceived importance and willingness to use such an instrument among surveyed guideline writers
- Our findings identify a gap in guidance around a key guideline development task and propose a potential solution

## Background

Clinical practice guidelines (CPGs) are developed through a rigorous process of evidence evaluation with the aim of facilitating the implementation of evidence and standardizing best practices among practitioners [1]. However, these goals are often not realized due to a variety of constraints categorized as either extrinsic (focused on the external practice environment) or intrinsic (focused on the guidelines themselves). Specifically, extrinsic factors focus on provider and patient knowledge, motivation, and skill, and system-level constraints that include the organizational context, provider workflow and practice environment. Intrinsic factors, on the other hand, refer to inherent features associated with the guidelines themselves (such as the content, formatting, and length) [2].

“Implementability” of a CPG refers to a set of guideline characteristics that predict how effectively that CPG can be implemented [3, 4]. Although both intrinsic and extrinsic factors are important when seeking to strengthen guideline implementation, many scholars have argued that a focus on improving the intrinsic quality may be a more cost-effective and broadly applicable approach [2]. To this end, Kastner and colleagues conducted a comprehensive realist review to define and describe the intrinsic attributes of guidelines that impact their implementability. These findings were then refined and validated through an iterative consensus process involving 248 guideline experts from 34 countries, to produce the Guideline Implementability for Decision Excellence Model (GUIDE-M) [5]. This model describes three core areas that influence guideline implementability: (1) the “developers” of guideline content (addressing comprehensive representation, knowledgeable and credible developers, and management of competing interests); (2) “creating” content (addressing evidence synthesis and deliberations and contextualization); and (3) “communicating” this content (addressing the language and the format used to present messages) [5].

Existing widely used tools for creation of guidelines (“guidelines for guidelines”) address many of the identified domains, in particular those pertaining to “developers” and “creating” content. However, despite that effective communication through language and format optimization has been associated with greater uptake [4, 6], this third pillar of guideline implementability has not been the specific focus of any existing tools [5].

To address this gap, our overall aim was to develop a tool that can be used by guideline developers to optimize language and format during guideline development, thereby enhancing guideline uptake. In this study, we sought to: develop a prototype of this tool; evaluate for any comparable guidance available in existing resources; and evaluate the perceived importance of included concepts and need for such a tool among guideline developers. Herein, we report content development for the language and format instrument, including identification and organization of language and format constructs to be included followed by face validation of an instrument prototype (phase 1); identification of guidance pertaining to language and format constructs in existing guideline tools (phase 2); and evaluation of language- and format-related preferences and perceived importance and need for such an instrument in an internationally representative group of guideline developers (phase 3).

## Methods

We used a mixed-methods design to address our objectives, consisting of three iterative phases: (1) content development for the prototype tool, called the Guideline Language and Format Instrument (GLAFI), to identify candidate domains for inclusion; (2) document analysis of existing guideline tools to catalog currently available language and format guidance, including missing items, new items, and overlap between existing tools; and (3) an international survey of guideline developers, eliciting their perceptions of the importance of language and format concepts, the quality of existing resources to address these concepts, and usefulness of a language and format tool.

### Phase 1: content development for a guideline language and format instrument

- ***Identification and organization of language and format constructs for inclusion in the tool***

In order to identify candidate domains for inclusion in a guideline language and format tool, we started by extracting all attributes in the “Communicating content” tactic in the GUIDE-M implementability framework [5]. We further complemented this list with all language and format attributes and sub-attributes presented in Kastner and colleagues’ 2015 realist review [2] (which contained more detailed sub-domains than the GUIDE-M). Our goal was to fashion these attributes as actionable constructs that may facilitate implementability. Guideline experts MK and SG reviewed this comprehensive list independently to identify all constructs that could be included in a language and format tool. Criteria for inclusion were (1) evidence exists that adhering to the practice represented in the construct improves uptake of the content; (2) feasible to explain to non-expert guideline developers through description and/or an example; (3) feasible for non-expert guideline developers to determine whether existing content adheres to the practice recommended in the construct (for assessment of existing guideline content); (4) actionable, either to improve existing content or when being considered during de-novo content production; (5) feasible for non-expert guideline developers to understand and address with minimal or no training or external guidance; and (6) distinct from direction typically provided in the process of journal typesetting (relevant for format-related concepts) (e.g., journals often have established conventions for format issues such as how subtitles are presented). All discrepancies were resolved through discussion, and the final list was vetted by a 3rd guideline expert (IF). Based on this comprehensive list of constructs and in accordance with the hierarchy presented in the referenced documents, we then created the following *items*: *domains* (global categories), *subdomains* (sub-categories within each domain), and *action items* (individual actionable recommendations with explanatory operational definitions and examples). Referring back to the realist review [2], and to original literature sources where required, we drafted a description, including both the definition and the evidence-based expected benefit of adhering to that practice, for each domain and subdomain. These items were then organized into a prototype tool.

b) ***Face validation of language and format items***

The prototype tool was then presented to a group of 18 guideline experts participating in the annual Canadian Thoracic Society (CTS) Guideline Methodology Workshop (Vancouver, British Columbia, April 2018). This study was approved by the North York General Hospital Research Ethics Board (REB# 18-0008), and all participants provided written informed consent. Two members of our research team (SG and MK) led a 2-h workshop to introduce the prototype tool and to test its face validity. The session began with a didactic presentation on the importance of and evidence for language and format concepts, and an introduction to the prototype tool. Next, participants were organized into 4 small groups (4–6 individuals per group, with diverse guideline development experience, roles, and expertise, and representation from both organizations, where possible). To test the face validity of the prototype, each group was asked to apply the paper-based prototype tool on 4 specific guideline recommendation examples from recent CTS (3) or Chest (1) guidelines (Additional file 1). The goal for each small group was to optimize the language in each guideline recommendation by using the tool to identify and address language concerns. At the conclusion of the small-group work, the moderators reviewed the language issues identified for all recommendations with the entire group of workshop participants, presented a proposed revised version for each, and solicited feedback (recommendations and suggestions for content and usability improvements) on the items and the overall prototype tool.

At the conclusion of the workshop, consenting participants completed an anonymous paper-based evaluation survey capturing demographic information and perceived usefulness of the prototype tool, including a Likert scale ranking the usefulness of each action item (individual actionable recommendations within domains). Any action item with a mean Likert scale usefulness rating of < 4/5 was re-structured in the prototype tool (i.e., the description and/or accompanying example were re-drafted). Lead authors (MK, SG) also assessed all open-ended feedback in the questionnaire and made corresponding improvements to the structure, descriptions, and content of included elements.

### Phase 2: document analysis of existing guideline tools

Next, we used a document analysis approach to identify guidance pertaining to language and format constructs in existing commonly used guideline tools/approaches (“tools”) [7]. We selected tools that were identified by the GUIDE-M group for comparative analysis, as per the following criteria applied by GUIDE-M: (i) published or unpublished reports freely available in the public domain; referenced in the realist review of guideline implementability domains [2]; (ii) designed to provide practical advice related to guideline development, reporting or appraisal; and (iii) perceived by experts to be in wide use internationally [5]. The original list of guideline tools that met all of these criteria were: AGREE II [8], IOM standards [9], the Guideline International Network (G-I-N) standards [10], Guidelines 2.0 [11], ADAPTE [12], and GRADE [13]. The GUIDE-M group also added the GLIA instrument [3], as it specifically addresses guideline implementability. In our assessment of their eligibility, we eliminated the ADAPTE tool [12], since it focuses on adaptation of existing guidance for a specific context, rather than de novo guidance development; and added the AGREE-REX tool [14], which was developed in response to a gap identified in the GUIDE-M analysis, and which our research team perceived to be an emerging tool of importance in optimizing guideline credibility and implementation.

To perform our document analysis, we identified the most recent and most readily available version of each guideline tool (i.e., the version most likely to be used by guideline developers, as opposed to derivative or explanatory publications). Two reviewers (SG and KP) independently analyzed each tool to identify any language and format guidance, including advice that matched any existing items in our prototype tool. We also sought to identify any new concepts. Using an Excel^TM^ spreadsheet containing each item of our prototype tool, each reviewer worked independently to identify and match elements of the existing guideline tools with those within the language/format constructs in our tool, adding specific quotes and page references next to the identified item.

For each of our language and format items that were identified within the guideline tool, reviewers further qualified the nature of the guidance by classifying the item as either (i) *mentioned* in the guideline tool (alluded to without description); (ii) *described* in the guideline tool (provided a description and /or explanation of the item, with or without a rationale, but without guidance on how a guideline developer would operationalize it in practice); or (iii) operationalized in the guideline tool (provided sufficient detail for a guideline developer to take action and apply the item in their guideline writing/formatting). These independent analyses were reviewed by a 3rd reviewer (MK), and any discrepancies resolved by discussion and review of the original guideline tools. A descriptive summary of findings included the proportion of tools that mentioned, described, and/or operationalized each item (the denominator used for total items was all action items + any domain/subdomain we found addressed in an existing tool), and the proportion of items that were mentioned, described, and/or operationalized in each existing tool. For any newly identified language or format domains, sub-domains, or action items from existing guideline tools, we included these in our prototype if they met any of the following pre-set criteria: evidence for effect on uptake of content; recommendation found in more than one existing guideline tool; or consensus among research team members that the element adds practical value for the target user of the guideline (e.g., improving efficiency of consuming guideline information) without apparent deleterious consequences.

### Phase 3: International survey of guideline developers

Next, we conducted a survey with an internationally representative group of guideline developers to measure perceptions of the importance of language and format items and the adequacy of existing resources to address these items, and the potential usefulness of a targeted tool addressing these issues. The study was approved by the North York General Hospital Research Ethics Board (REB# 18-0008), and all participants provided written informed consent.

We aimed to recruit a broadly representative sample of both Canadian and international guideline developers. To identify target participants, we searched for English language guidelines indexed in the: Canadian Medical Association’s Joule Clinical Practice Guideline (CPG) Infobase (a database of over 1200 recent (last 2 years) evidence-based, rigorously produced guidelines developed or endorsed by authoritative medical or health organizations in Canada) [15] (January 2017–July 2019); and the Guidelines International Network (G-I-N) International Guideline Library (a library of over 6500 guidelines developed or endorsed by organizations around the world) [16] (January 2014–July 2019). For each unique guideline, we retrieved the original guideline publication and documented the corresponding author’s email address. Where a corresponding author or their contact information could not be identified, we searched for the email address of the first author, last author, committee chair, or committee co-chair (in this order of preference).

We emailed each identified author to provide a brief introductory background and a link to a ~ 10 min survey (SurveyMonkey^TM^) (September 2019). To maximize response rates, we sent non-responders a reminder email 2 weeks after the original email and remaining non-responders another email 1 week after that. For any undeliverable email addresses, we attempted to identify alternative contacts for authors from the same guideline publication, applying the same priority as that noted above.

**Fig. 1:**
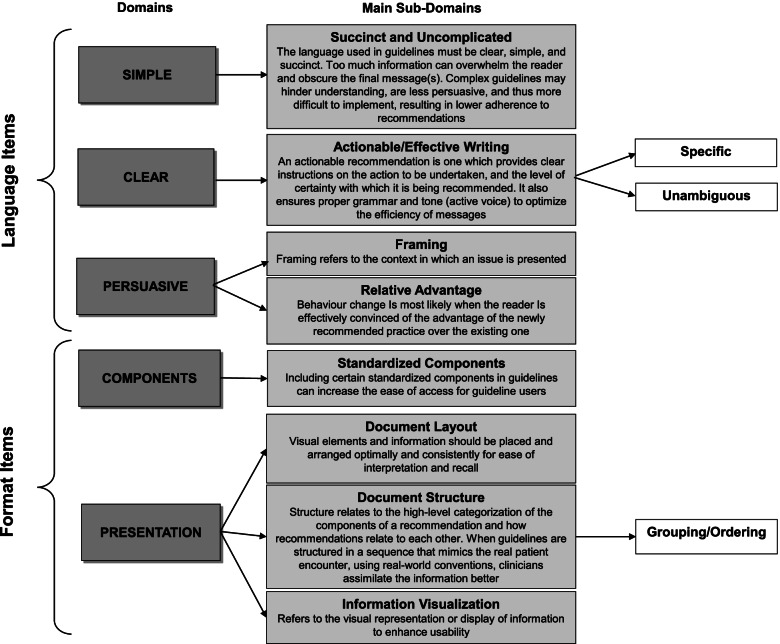
Language and format tool organizational structure into domains and main subdomains

The survey was developed iteratively by authors SG, MK, and RT, with serial edits based on pilot testing and feedback from 3 external guideline experts on questionnaire content, clarity, and length. The survey described the concept of the tool, prior work, and definitions of the 4 main proposed subdomains under “language” and “format” (definitions available in Fig. 1). It included Likert-scale and open-ended questions, and aimed to capture respondent: (i) demographics; (ii) perceptions of the guidance provided by existing guideline tools (specifically assessing the tools included in phase 2), across each language and format subdomain in our prototype tool; (iii) importance rankings of each subdomain in our prototype tool; (iv) perceptions of the importance of considering language and format items on end-user uptake of guideline recommendations; and (v) likelihood that their guideline development organization would adopt a targeted language and format tool in their guideline production process. We invited respondents to indicate any additional tools/approaches used by their organization that were not among the tools included in our phase 2 analysis, and planned to add any tool used by ≥ 10% of responding organizations to our phase 2 analysis. Quantitative survey responses were analyzed using descriptive statistics (frequencies, means and standard deviations).

## Results

Our results are reported according to each of the three phases of our inquiry.

### Phase 1: content development for a language and format tool

- ***Identification and organization of language and format constructs for inclusion in a guideline language and format tool***

**Fig. 2:**
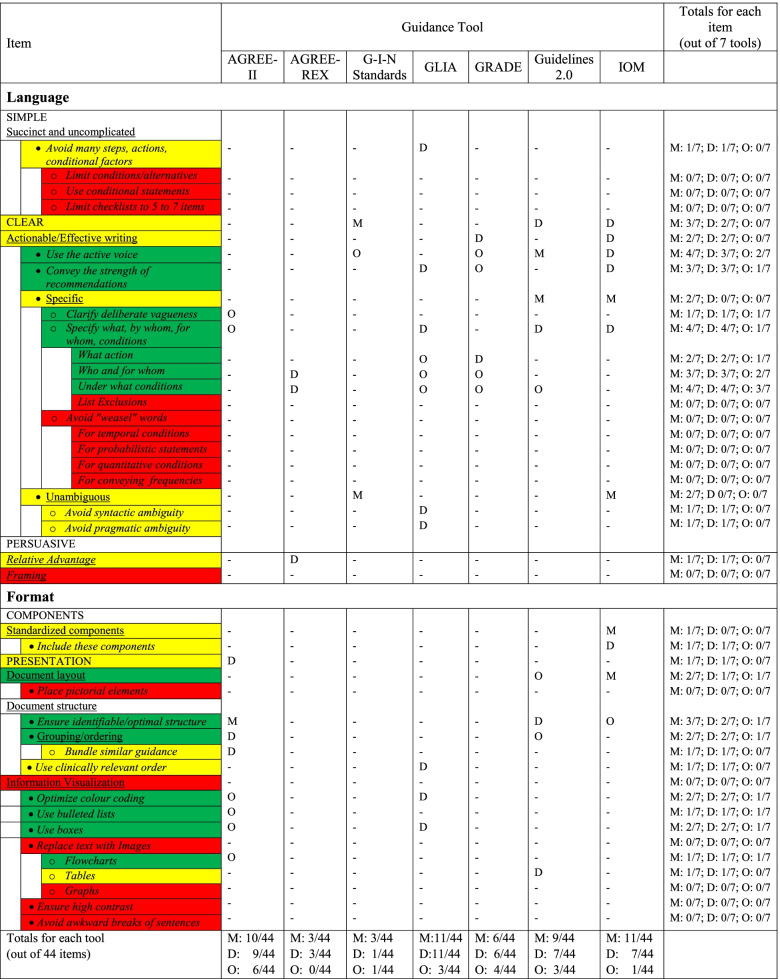
Language and format action item coverage in existing guidance tools**.** Constructs meeting inclusion criteria were organized into the following *items*: *domains* (global categories), *subdomains* (sub-categories within each domain), and *action items* (individual actionable recommendations with explanatory operational definitions and examples). Domains are capitalized; sub-domains are underlined; and action items are italicized (note that some sub-domains were also considered action items). Action items that were operationalized in at least 1 tool are shaded green, those that were either mentioned or described in at least 1 tool are shaded yellow, and those that were neither mentioned, described, nor operationalized in at least 1 tool are shaded red (items are ordered green/yellow/red where applicable, within each category). M denotes mentioned; D denotes described (implies that the item was also mentioned); O denotes operationalized (implies that the item was also mentioned and described)

**Fig. 3:**
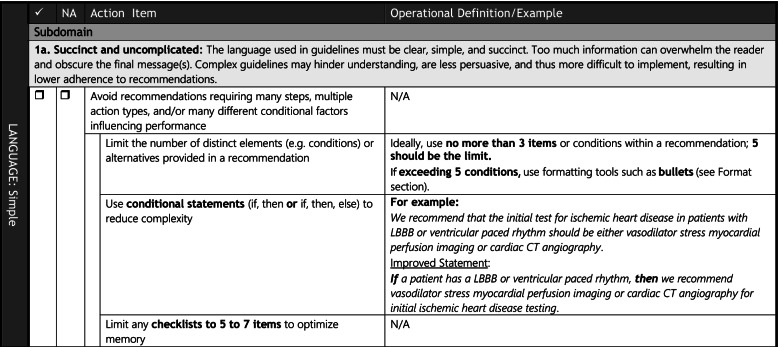
Example of construct organization into domains, subdomains, and action items in the GLAFI. Under the global “LANGUAGE” category and “Simple” domain, a main subdomain was called “Succint and uncomplicated.” Under this subdomain were 4 action items, including “Avoid recommendations requiring many steps … ” and the following distinct items under that category: “Limit the number of distinct elements … ”; “Use conditional statements … ” and “Limit any checklists to 5 to 7 items … ”

Constructs which met inclusion criteria were organized into 3 main language domains with 4 subdomains (21 action items) and 2 main format domains with 4 subdomains (14 action items) (Figs. 1 and 2). An example of a sub-domain definition and corresponding action items in the tool is provided in Fig. 3.

b) ***Face validation of language and format items***

All 18 guideline experts participating in the CTS Guideline Methodology Workshop agreed to participate in the study. The group included 17 clinicians and 1 guideline methodologist involved in guideline production at the CTS (15) and Chest (3). Participants were aged 40–49 (*n* = 7), 50–59 (*n* = 8), or over 60 years (*n* = 2), and the majority were female (*n* = 10) and worked in an academic setting (*n* = 15). Among the 17 physicians, 14 had been in practice for at least 15 years. On the anonymous feedback survey, all participants perceived (i.e., indicated a Likert scale score of 5/6/7 on a 7-point scale, with a mean score of 6.1/7) that the tool would improve the implementability of guideline recommendations. Although half perceived that it would significantly slow down the guideline production process (mean Likert score 4.3/7), almost all (14/15, 93%; mean Likert score 5.9/7) indicated that it should be used by all future CTS guideline panels. By the end of the session, 12/17 (71%) participants believed that they had adequate knowledge and expertise to improve the language and format of their guideline recommendations through use of this tool.

Five (14%) of the 35 identified action items (2 language items, 3 format items) received a mean Likert scale importance rating of less than 4 out of 5 and were re-structured (collapsed into existing items or re-worded) after study completion (Likert-scale scores for each action item are provided in Additional file 2). Based on global feedback received during the session, the tool was also divided into more clearly distinct language and format sections, and we provided additional examples explicating action items, where possible.

### Phase 2: document analysis of existing guideline tools

Of 25 language items (21 pre-defined action items + 4 domains/sub-domains we found mentioned in existing tools), 15 items (60%) were mentioned in at least one tool, 13 items (52%) described in at least one tool, and only 7 items (28%) operationalized in at least one of the seven existing guideline tools in our analysis (Fig. 2). Of 19 format items (14 pre-defined action items + 5 domains/sub-domains we found mentioned in existing tools), 13 items (68%) were mentioned in at least one tool, 12 items (63%) described in at least one tool, and only 7 items (37%) operationalized in at least one tool (Fig. 2). Accordingly, 10 of the 25 language items (40%) and 6 of the 19 format items (32%) were not mentioned (and by extension not described or operationalized) in any of the seven existing guideline tools. The pre-existing guideline tool that addressed (i.e., at least mentioned) the most language items was the GLIA (8/25–32%), the most format items was the AGREE-II (8/19–42%), and the most overall items were the GLIA (11/44–25%) and IOM (11/44–25%).

Based on our analysis of the existing guideline tools, our data allowed us to add 1 new subdomain and 5 net new action items pertaining to “language” (we added 3 new items under the new subdomain and replaced 1 existing item with 3 more detailed items under an existing subdomain) (Table 1). We did not add any new subdomains or action items pertaining to “format” but added to the existing operational definition for 1 action item (Table 1). The final tool is presented in Additional file 3.

**Table 1:** Updates to language/format items after analysis of existing guidance tools

Subdomain (underlined)	Original content	Updated content	Source and justification
**Language** CLEAR actionable/effective writing	Action item: Use words that convey the **strength** of recommendations (as per GRADE guidelines)	Action items: If using the GRADE approach: Identify recommendations according to their **strength** Use an **action verb** corresponding to the strength of a recommendation to operationalize it Employ **consistent use of a letter, number, and/or symbol system** for characterizing both the strength of a recommendation and the quality of evidence	Source: GRADE Handbook [13] Justification: authors’ consensus
**Language** CLEAR consistent use of terms^a^	N/A	Action items: Use the same ***semantic indicators*** (use the same terminology to indicate level of evidence, strength of recommendation, and the action verbs) across recommendations When comparing alternative approaches, always ***frame the recommendations*** in favor of a particular management approach rather than against an alternative ***Reserve use of “not”*** for recommendations against a management approach that may be particularly harmful and/or widespread	Source: IOM [9] Justification: author consensus Source: GRADE Handbook [13] Justification: author consensus Source: GRADE Handbook [13] Justification: author consensus
**Format** Presentation document structure	Action item: Ensure that the guideline has a **clearly identifiable and optimal structure** **Operational definition:** • Clear chunking (grouping) of information: Use sequential arrangement or bundling • Ensure standardized usage of formatting indicators such as type sizes and weights (e.g., bold) • Consider structuring by dividing patients into specific subclasses, if relevant	Action item: Ensure that the guideline has a **clearly identifiable and optimal structure** **Operational definition:** **- Newly added components**: • Group specific recommendations near the summary of key evidence for those recommendations • Consider using bold and/or underline to draw attention to all recommendations, or, if applicable, to a subset of recommendations pertaining to the main PICO question(s) covered by the guideline • Report recommendations in a way that is visible and easy to find (i.e. do not embed recommendations within long paragraphs, and consider grouping recommendations in a summary section).	Source: AGREE II [8] Justification: authors’ consensus Source: AGREE II [8], GLIA [3] Justification: found in more than one existing guidance document Source: IOM [9], GUIDELINES 2.0 [11], AGREE II [8] Justification: found in more than one existing guidance document

^a^Newly added subdomain, defined as: Ensure that the same terms are used across recommendations whenever possible, and that these terms are used to consistently (i.e. to indicate the same meaning). Concepts within this subdomain were identified in 3 reference guidance documents: GRADE, IOM, Guidelines 2.0

### Phase 3: international survey of guideline developers

**Fig. 4:**
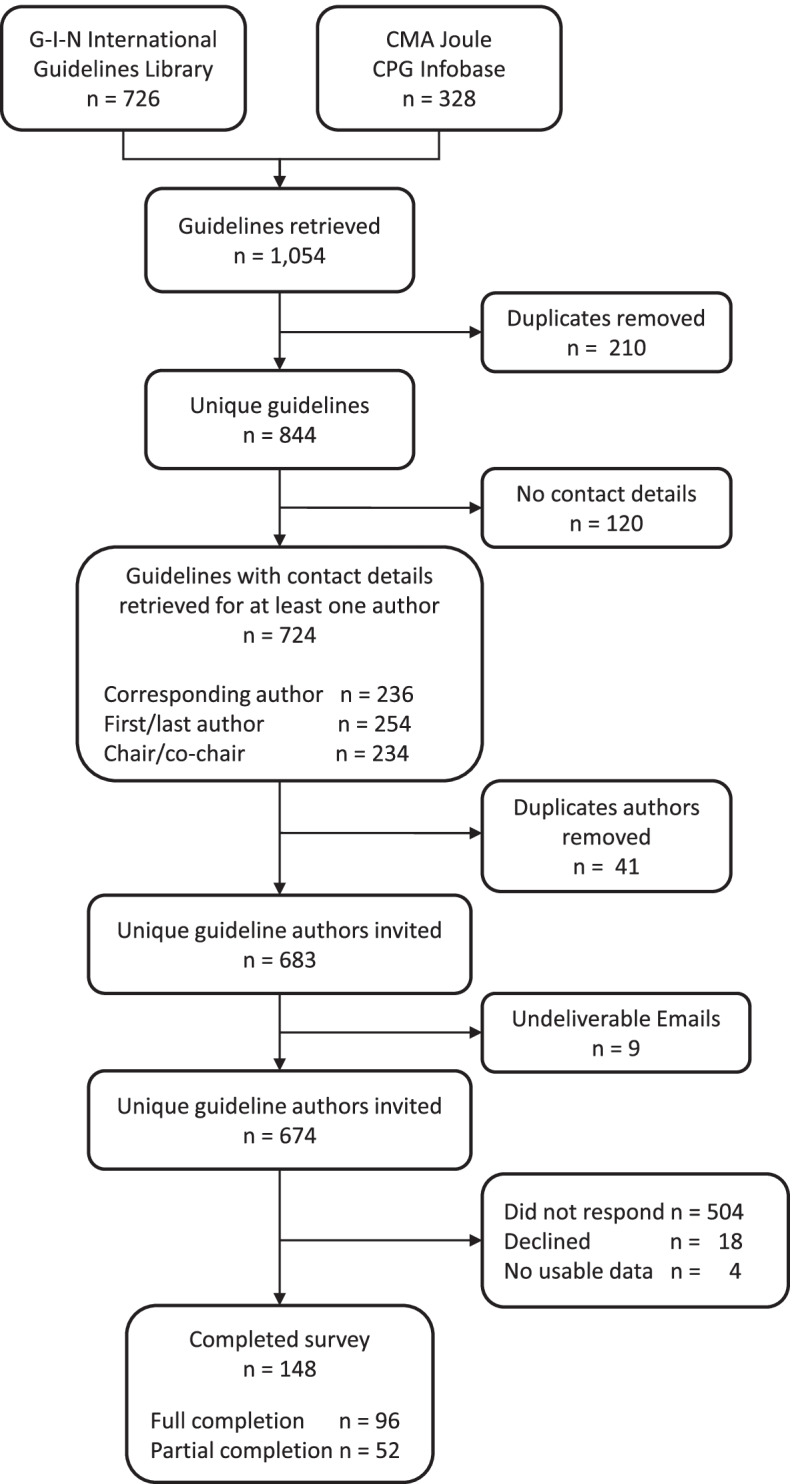
Flowchart of survey respondents

We identified 1054 unique clinical practice guidelines from the CPG Infobase (*n* = 328) and G-I-N International Guidelines Library (*n* = 726). Among these, 210 (20%) guidelines were duplicates and 120 (11%) guidelines had no available author contacts, leaving 724 (69%) guidelines for which a contact email address was available [corresponding authors (33%); first or senior authors (35%); and guideline chair and/or co-chairs (32%)]. Further removal of 41 duplicate authors resulted in the final sample of 683 unique guideline developers (representing 724 identified guidelines) who were invited to complete the survey via email. Nine email addresses (1.3%) were invalid, and replacements could not be found. Among the remaining 674 unique eligible guideline authors, 18 (2.7%) declined to participate (one of these provided an alternate contact who did complete it), 4 (0.6%) provided no usable data, and 148 responded, for a response rate of 22.0% (Fig. 4).

#### Characteristics of survey respondents

Survey respondent characteristics are described in Table 2. Respondents produced guidelines pertaining to medicine (76.4%), surgery (20.3%), and allied health care (3.4%), representing 9 countries and 45 different organizations, and reported a mean of 11.6 (SD 7.2) years of guideline development experience. Although the median number of guidelines represented in our sample was one per organization, 5 organizations had 10 or more guidelines represented: the National Institutes of Health and Care Excellence (21); Diabetes Canada (19); the Society of Obstetricians and Gynaecologists of Canada (16); Cancer Care Ontario (14); and the American Society of Clinical Oncology (13).

**Table 2:** Characteristics of guideline developer survey respondents (*n* = 148)

Characteristic	***N*** (%)
Sex	
Female	72 (48.6)
Male	76 (51.4)
Age	
31–40 years	14 (9.5)
41–50 years	50 (33.8)
51–60 years	47 (31.8)
> 60 years	37 (25.0)
Geographic background	
Canada	91 (61.5)
UK	21 (14.2)
USA	20 (13.5)
Other^a^	16 (10.8)
Number of guidelines previously developed	
1–2	28 (18.9)
3–5	56 (37.8)
6–9	26 (17.6)
≥ 10	38 (25.7)
Roles played in prior guideline development^b^	
Chair/leader	124 (83.8)
Deciding on methods	76 (51.4)
Selecting question	110 (74.3)
Searching the literature	88 (59.5)
Reviewing evidence	132 (89.2)
Appraising evidence	127 (85.8)
Synthesizing evidence	112 (75.7)
Formulating recommendations	141 (95.3)
Planning guideline dissemination/implementation	96 (64.9)

^a^Included: Australia, Belgium, Denmark, Europe (International), Malaysia, and Saudi Arabia

^b^Roles categories were not mutually exclusive

#### Characteristics of guideline organizations and tools

Among the 45 guideline organizations represented in our sample, the proportion currently using each of the seven selected guidance tools was: GRADE (66.7%); AGREE II (33.3%); IOM Standards (20.0%); GIN Standards (6.7%); Guidelines 2.0 (4.4%); AGREE-REX (0%); GLIA (0%). All guideline organizations used at least one tool. During the study period, none of the organizations reported using the more recent RIGHT [17] or GRADE-ADOLOPMENT [18] tools. Six of 45 (13.3%) represented organizations also indicated that they use other tools; however, no tool was used by ≥ 10% of responding organizations. Only the National Institutes of Health and Care Excellence (NICE) tool was used by more than one group [NICE (2/45–4.4%); GuideLines Into DEcision Support (GLIDES) (1/45–2.2%); deprescribing guideline methods from Farrell, et al. [19] (1/45–2.2%); The Canadian Task Force on Preventive Health Care methods (1/45–2.2%); and Diabetes Canada guideline methods (1/45–2.2%)].

**Fig. 5:**
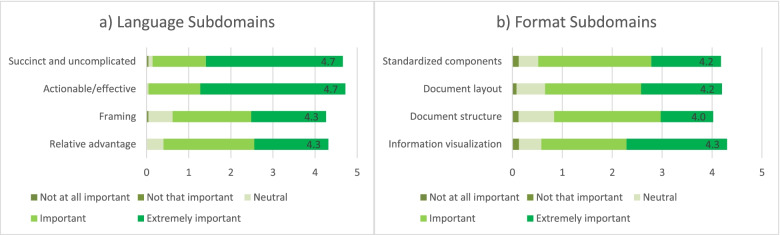
Survey respondent (guideline developer) ratings of the importance of main language (**a**) and format (**b**) subdomains for recommendation uptake. Guideline developer ratings of the importance of main language (**a**) and format (**b**) subdomains for recommendation uptake, in the GLAFI. The mean Likert scale response (out of 5) for each question is represented by the length of the bar and stipulated numerically within the bar. The proportion with each response type is represented by corresponding colors within each the bar

Table 3 shows respondents’ perceptions of whether existing guidance tools provide explicit guidance related to each main language and format subdomain in the GLAFI. Overall, the language used in guideline recommendations was rated as “extremely important” or “important” in determining end-user uptake by 90/96 (93.8%) respondents, and the format by 81/96 (84.4%). Correspondingly, 69/96 (71.9%) and 67/96 (69.8%) respondents indicated that their organization would be likely to use a dedicated tool for language and for format, respectively. Likert scale rankings for importance each main subdomain in determining recommendation uptake are depicted in Fig. 5.

**Table 3:** Guideline developer perceptions of explicit guidance on language and format provided in existing guidance tools

	Users’ report of explicit guidance provided in each tool						
	AGREE II	AGREE-REX	IOM	G-I-N	Guide-lines 2.0	GLIA	GRADE
**Language subdomains in the GLAFI**							
*N*^a^	50	4	19	11	10	4	78
Succinct and uncomplicated	21 (42%)	2 (50%)	7 (36.8%)	3 (27.3%)	4 (40%)	3 (75%)	36 (46.2%)
Actionable/effective writing	15 (30%)	2 (50%)	6 (31.6%)	4 (36.4%)	2 (20%)	4 (100%)	41 (52.6%)
Framing	13 (26%)	1 (25%)	7 (36.8%)	2 (18.2%)	0	0	32 (41.0%)
Relative advantage	9 (18%)	0	6 (31.6%)	2 (18.2%)	0	1 (25%)	26 (33.3%)
Standardized components	16 (32%)	0	7 (36.8%)	3 (27.3%)	4 (40%)	0	29 (37.2%)
Document layout	10 (20%)	0	6 (31.6%)	2 (18.2%)	3 (30%)	0	20 (25.6%)
Document structure	9 (18%)	0	4 (21.1%)	3 (27.3%)	2 (20%)	0	23 (29.5%)
Information visualization	7 (14%)	0	6 (31.6%)	1 (9.1%)	3 (30%)	0	19 (24.4%)

*AGREE* Appraisal of Guidelines Research and Evaluation, *REX* Recommendation EXcellence, *IOM* Institute of Medicine, *G-I-N* Guidelines International Network, *GLIA* GuideLine Implementability Appraisal, *GRADE* Grading of Recommendations, Assessment, Development and Evaluations

^a^The number of respondents who indicated a familiarity with each tool was used as the denominator for each subdomain response

## Discussion

In this mixed-methods study, we used existing evidence to develop a prototype tool—the Guideline Language and Format Instrument (GLAFI) - and demonstrated that it was usable and acceptable to guideline-writers in a face validation process, that existing guidance tools do not address most of the constructs it includes, and that international guideline developers ascribe a high importance to included constructs, along with a high level of willingness to use such a tool.

Over the past decade, a growing body of literature has emphasized the importance of simplifying the language and format in CPGs as a way to maximize user uptake [3, 20]. Clinicians report that CPGs are too lengthy, ambiguous, and complex [21–23], and characterize the primary barriers and facilitators to guideline uptake as a function of their format, language, and usability [24]. Qualitative studies demonstrate that guideline writing style is a key determinant of whether guidelines are followed [25], and poor guideline design can result in inappropriate clinical decisions [26]. Individual CPG attributes such as increased recommendation specificity and actionability have both been found to increase appropriate ordering and decrease inappropriate ordering [27], while a better writing style has improved user attitudes towards and intentions to implement guidelines[28]. At the same time, vague and imprecisely defined recommendations strongly predict guideline non-adherence [29]. Such findings were reinforced in Gagliardi and colleagues’ conceptual framework for guideline implementability, which specifically identified elements related to guideline *format* as providing valuable opportunities for improved uptake [4, 6].

Given the objective evidence of their impact on end-user uptake, we based the constructs represented in our prototype tool on those in the Language and Format domains in Kastner’s review [2] and the GUIDE-M framework [5]. We then analyzed seven existing guideline tools selected on the basis of objective criteria, including prior expert consensus that they are widely used internationally. Our analysis revealed major gaps in guidance surrounding language and format requirements for intrinsic implementability. Of 44 items, 17 (39%) were neither mentioned, described, nor operationalized in any of these existing tools. Furthermore, even when included, most concepts were simply mentioned and/or described, with only 14/44 (32%) actually operationalized (providing sufficient detail for a guideline developer to apply in practice), across all tools. No single tool *mentioned* even half of the recommended language or format items, and the best performing tools mentioned only one quarter of overall items. These findings suggest the existence of an important gap in providing guideline developers with guidance surrounding this core element of intrinsic implementability.

This gap may be explained by the fact that existing tools were primarily designed to address methodological and reporting concerns, and principally informed by the medical literature [20]. In contrast, constructs identified in the realist review drew on a wider range of disciplines focused on changing human behavior, including social, cognitive, and health psychology; marketing; business/management; and human-factors engineering literatures [2]—yielding novel insights into optimizing language and format. For example, human factors engineering literature reveals the importance of structuring guidelines to mirror end users’ work processes and approaches to care [30]. Marketing literature provides unique guidance for achieving persuasive and clear messaging [31], whereas design literature outlines design principles which improve the usability and attractiveness of products. Cognitive psychology further alerts to the limitations of information processing and provides explicit strategies for developers to ease guideline users’ cognitive load [2, 32].

We complimented this document analysis with a needs assessment in an internationally representative sample of guideline developers, representing 45 guidance-producing organizations. Developers spanned a wide range of medical disciplines and were highly experienced, having played a variety of roles in prior guideline development (Table 2). Their responses indicated a clear recognition of the overall importance of language and format for guideline uptake, along with high importance ratings for each main subdomain in our prototype tool. We noted that use of existing tools is eclectic across settings, with only the GRADE (67%) and AGREE II (33%) instruments in use by even one third of organizations. Yet, for 7 of the 8 main subdomains in our tool, a majority of experienced GRADE and AGREE users reported that these tools lacked any explicit guidance related to these concepts (Table 3). The tools which we found to include the most items—the GLIA and IOM Standards—were currently in use by 0 and 20% of these organizations, respectively.

A large number of guideline guidance tools are already in existence, whereby adding another tool raises concerns about duplication. However, no existing tool was specifically designed to address the “communicating content” “tactic” in the GUIDE-M Model [5], as confirmed in by our document analysis demonstrating gaps in existing tools. This suggests minimal overlap and significant added value from the GLAFI. However, concerns about guideline developer fatigue over new tools and requirements remain. Our tool prototype was face validated with guideline developers in an in-person hands-on workshop, ensuring end-user input as part of the development process, which enhances uptake [33]. This also demonstrated the practical feasibility of rendering naïve users comfortable with the tool in a single 2-h session (such a session can easily be provided as an online module, as are commonly in use for training with other tools) [34, 35]. We also confirmed that guideline developers perceived these concepts to be important, with each of the 8 main subdomains in our tool being rated important to extremely important to recommendation uptake. Most importantly, ~ 70% of respondents reported an organizational willingness to adopt a tool such as the GLAFI in their guidance development process. Still, the fact that a higher percentage of respondents acknowledged the importance of language and format constructs (94% and 84%, respectively) versus an organizational willingness to use a language or format tool (72% and 70%, respectively), likely indicates that there are barriers to use of such a tool that require further exploration. Practically, rather than having each guideline committee within an organization manage language and format requirements, we believe that larger guideline organizations might benefit from having an expert “Language/Format Team” which applies the tool with individual committees, vetting and editing each recommendation before voting, and the entire document before finalization, across guidelines.

Our study has several limitations. We developed a prototype tool grounded in a strong evidence base [2, 5] and complimented it with a formal document analysis of existing guidance tools, representing constructs associated with likelihood of implementing a recommendation. However, we recognize that given the diverse nature of the underlying scientific literatures that informed this evidence base [2], not all constructs represented in our tool were shown to directly improve *guideline* implementation (i.e., many were proven in content areas other than guidelines). Given that language and format influence uptake through common cognitive processes, we believe that these constructs are likely to be generalizable across disciplines. Criteria established for initial inclusion of constructs in the prototype tool were subjective, given a lack of appropriate measurable criteria. We also recognize that language and format constructs specifically targeted to English-language guidelines may not be applicable in other languages. Similarly, given that the development team and survey respondents were primarily from high-resource settings, the GLAFI may not yet be generalizable to low-resource settings. Our survey response rate of 22% might also reflect a sample of guideline writers who have a disproportionate interest in guideline methodology. Our future work will address these issues by exploring the generalizability of the GLAFI to a wide range of CPGs and users. We also note that although most constructs have an empirical foundation, some formatting constructs were based on best practices and end-user preferences [2]. Although we are not aware of any such proof-of-effect studies for existing guidance tools, it would be beneficial to study the impact of use of the GLAFI on the perceived implementability of a set of guideline recommendations among actual target end-users.

Next, although we formally analyzed 7 existing guideline tools, there are numerous other tools in existence. However, no single other tool was used by more than 2/45 (4.4%) of guideline organizations in our survey, and we are not aware of a tool that specifically addresses language and/or format constructs in CPGs. Although our tool attempted to exclude typical journal-specific format requirements that are usually specified in the process of typesetting, for guidelines published in medical journals, we recognize that guideline developers might still not have direct control over some of the recommended formatting elements. However, neither journal editors nor typesetters would be expected to be familiar with all of the relevant formatting items presented in our tool, and we believe that it behooves guideline development groups to advocate for evidence-based formatting when their documents are published, given their vested interest in successful adoption. These principles can also be applied to the variety of written guideline dissemination tools that are commonly generated by guideline-producing organizations. We also recognize that increasing use of electronic formats for guideline consumption (distinct from the .pdf format recreations of “paper” guidelines) will affect format constructs in the future [9]. In these formats, the electronic interface can be leveraged to organize information into layers [36] that facilitate retrieval and consumption, and human factors engineering should be leveraged to optimize the user interface. Finally, there is a growing focus on the importance of using language that avoids stigmatizing, excluding, and/or marginalizing vulnerable groups [an Equity, Diversity and Inclusion (EDI) consideration]. Although not a current focus of the GLAFI, inclusion of guidance regarding this important area can be explored in future GLAFI development work.

## Conclusions

In summary, we present the multi-step development process leading to the prototype GLAFI tool, designed to help guideline developers to optimize the language and format of their guidelines in accordance with best evidence for optimal uptake. Our tool directly addresses a fundamental pillar of guideline implementability which has not yet been the focus of guideline tools, and which our analysis demonstrates is inadequately addressed in commonly used current tools. Our survey of international guideline developers confirms the perceived importance of these concepts, perceived lack of guidance in existing resources, and a willingness to adopt such a tool. Next, we plan to further refine the tool in serial qualitative focus groups with diverse guideline developers, before validating its effect on perceived guideline implementability with target stakeholders (i.e., clinicians). Ultimately, broad usage of such a tool will require awareness and recognition of the importance of language and format among guideline-producing organizations and guideline developers, to justify the additional time and resources for application of these principles in the guideline process.

## Supplementary Information

**Additional file 1.** Sample recommendations presented during face validation exercise.

**Additional file 2.** Likert-scale scores for each item (face validation stage).

**Additional file 3.** The Guideline Language and Format Instrument (GLAFI).

## Data Availability

Available upon request.

## Abbreviations

CPG: Clinical practice guideline
GUIDE-M: Guideline Implementability for Decision Excellence Model
GLAFI: Guideline Language and Format Instrument
CTS: Canadian Thoracic Society
REB: Research ethics board
G-I-N: Guidelines International Network
GLIA: GuideLine Implementability Appraisal
M: Mentioned
D: Described
O: Operationalized
SD: Standard deviation
