# Supplementary material for: The Guideline Language and Format Instrument (GLAFI): development process and international needs assessment survey
Source: Implement Sci. 2022 Jul 19;17:47. doi: 10.1186/s13012-022-01219-2 (PMC9295534; doi:10.1186/s13012-022-01219-2)
Supplement: Supplementary file 1 — Additional file 1. Sample recommendations presented during face validation exercise. [file 13012_2022_1219_MOESM1_ESM.docx]

**Additional File 1: Sample Recommendations Presented During Face Validation Exercise**

1. “In exacerbation-prone individuals 12 years of age and over with moderate asthma and poor control on a fixed-dose maintenance ICS/LABA combination, we suggest the use of budesonide/formoterol as a reliever be considered at the same maintenance ICS dose.”

Source: Lougheed MD, Lemiere C, Ducharme FM, Licskai C, Dell SD, Rowe BH, et al. Canadian Thoracic Society 2012 guideline update: diagnosis and management of asthma in preschoolers, children and adults. Can Respir J. 2012;19(2):144.

GLAFI items highlighted:

**Language**/SIMPLE/Succinct and uncomplicated/*Limit conditions/alternatives*

**Language**/SIMPLE/Succinct and uncomplicated/*Use conditional statements*

**Language**/CLEAR/Actionable/Effective writing/*Use the active voice*

**Language**/CLEAR/Actionable/Effective writing/Specific/*Specify what, by whom, for whom, conditions* ***(****what action, who)*

**Language**/CLEAR/Actionable/Effective writing/Specific/*Avoid weasel words (for quantitative conditions, for conveying frequencies)*

**Language**/CLEAR/Actionable/Effective writing/Unambiguous/*Avoid pragmatic ambiguity*

2. “Consider tracheostomy and HMV in children with bronchopulmonary dysplasia with stable pressure settings that are achievable with a home ventilator, with an FiO2 < 0.4 and children who are otherwise medically stable, demonstrating stable growth, and can be safely transported. (Grade 1C)”

Source: Amin R, MacLusky I, Zielinski D, Adderley R, Carnevale F, Chiang J, et al., & Canadian Thoracic Society. Pediatric home mechanical ventilation: A Canadian Thoracic Society clinical practice guideline executive summary, Canadian Journal of Respiratory, Critical Care, and Sleep Medicine. 2017;1(1):22.

GLAFI items highlighted:

**Language**/SIMPLE/Succinct and uncomplicated/*Limit conditions/alternatives*

**Language**/SIMPLE/Succinct and uncomplicated/Us*e conditional statements*

**Language**/CLEAR/Actionable/Effective writing/*Convey the strength of recommendations*

**Language**/CLEAR/Actionable/Effective writing/*Use the active voice*

**Language**/CLEAR/Actionable/Effective writing/Unambiguous/*Avoid syntactic ambiguity*

**Language**/CLEAR/Actionable/Effective writing/Specific/*Avoid weasel words (for quantitative conditions)*

3. “In patients with COPD with a previous or recent history of exacerbations, we recommend education and case management that includes direct access to a health- care specialist at least monthly to prevent severe acute exacerbations of COPD, as assessed by decreases in hospitalizations (Grade 1C).”

Source: Criner GJ, Bourbeau J, Diekemper RL, Ouellette DR, Goodridge D, Hernandez P, et al. Prevention of Acute Exacerbation of COPD: American College of Chest Physicians and Canadian Thoracic Society Guideline. Chest **2015;147(4):886**.

GLAFI items highlighted:

**Language**/CLEAR/Actionable/Effective writing/Unambiguous/*Avoid pragmatic ambiguity*

**Language**/CLEAR/Actionable/Effective writing/Specific/*Avoid weasel words (for temporal conditions)*

**Language**/CLEAR/Actionable/Effective writing/Specific/*Specify what, by whom, for whom, conditions* ***(****what action, who)*

4. “We suggest that in stable COPD patients who experience persistent dyspnea, exercise intolerance, and/or poor health status despite use of inhaled LAMA or LABA monotherapy that they be considered for treatment “step up” with LAMA plus LABA dual therapy (Grade 2A).”

Source: Bourbeau J, Bhutani M, Hernandez P, Marciniuk DD, Aaron SD, Balter M, et al. CTS position statement: Pharmacotherapy in patients with COPD—An update. Canadian Journal of Respiratory, Critical Care, and Sleep Medicine. 2017;1(4):228.

GLAFI items highlighted:

**Language**/CLEAR/Actionable/Effective writing/Unambiguous

**Language**/CLEAR/Actionable/Effective writing/Specific/*Avoid weasel words (for quantitative conditions)*

**Language**/CLEAR/Actionable/Effective writing/*Use the active voice*
