# Supplementary material for: The Guideline Language and Format Instrument (GLAFI): development process and international needs assessment survey
Source: Implement Sci. 2022 Jul 19;17:47. doi: 10.1186/s13012-022-01219-2 (PMC9295534; doi:10.1186/s13012-022-01219-2)
Supplement: Supplementary file 2 — Additional file 2. Likert-scale scores for each item (face validation stage). [file 13012_2022_1219_MOESM2_ESM.docx]

**Additional File 2: Likert-Scale Scores for Each Item (Face Validation Stage)**

| Item^a^ | Mean Likert Score^b^ |
| --- | --- |
| **Language** |  |
| SIMPLE  Succinct and Uncomplicated |  |
| *Avoid recommendations requiring many steps, multiple action types, and/or many different conditional factors influencing performance* | 4.8 |
| *Limit the number of distinct elements (e.g., conditions) or alternatives provided in a recommendation* | 4.3 |
| *Use conditional statements (if, then or if, then, else) to reduce complexity* | 4.1 |
| *Limit any checklists to 5 to 7 items to optimize memory* | 4.0 |
| CLEAR  Actionable/Effective Writing |  |
| *Use the active voice to make instructions more actionable* | 4.3 |
| *Use words that convey the strength of recommendations* (as per GRADE guidelines)  Specific | 4.2 |
| *Specify the target behaviour or action that needs to be performed, by whom, for whom, and under what conditions* | 4.6 |
| *What precise action is to be performed (define a measureable, recordable action using action-type verbs)* | 4.3 |
| *Who is responsible for completing the action in a given recommendation and for whom* | 3.3 |
| *State under what specific conditions or circumstances (e.g., when and where) the action is to be performed* | 4.3 |
| *List exclusions: circumstances where the action should not be performed* | 4.2 |
| *Avoid weasel words* (vague and under-specified words or phrases) | 4.2 |
| *For conveying temporal conditions* | 4.2 |
| *For conveying probablistic statements* | 4.2 |
| *For conveying quantitative conditions* | 4.3 |
| *For conveying frequencies, maximum and minimum values* (ceilings and floors) | 4.3 |
| *If ambiguity or vagueness is created deliberately (deliberate vagueness), provide an explicit statement acknowledging the vagueness and state the reasons* | 4.6 |
| Unambiguous  *Avoid syntactic ambiguity* (ambiguity caused by the structure of the syntax such as lack of punctuation, especially when using Boolean connectors) | 4.6 |
| *Avoid pragmatic ambiguity* (when guidance is not pragmatic because two or more recommendations within a guideline conflict with one another or a recommendation does not include instructions for common clinical scenarios) | 4.7 |
| PERSUASIVE  *Framing: when justifying a recommendation, place emphasis on the beneficial outcomes to be gained from adopting the recommendation* | 4.0 |
| *Relative Advantage: when a recommendation calls to change a previous, established practice, conceptualize the benefits of the new practice over the previous one in multiple domains* | 3.9 |
| **Format** |  |
| COMPONENTS  Standardized Components  *Ensure that the following components are included in the guideline…* | 4.3 |
| PRESENTATION  Document Layout  *Place pictorial elements (e.g., tables / graphs/ flowcharts) on the left-hand-side of documents and text on the right*  Document Structure | 3.9 |
| *Ensure that the guideline has a clearly identifiable and optimal structure* | 4.7 |
| *Present information in an expected and clinically relevant order* (match the guideline to the real world) | 4.8 |
| Grouping/Ordering |  |
| *When a single recommendation or set of recommendations contains many different types of guidance, bundle similar types of guidance together* | 4.6 |
| Information Visualization |  |
| *Replace textual explanations with images that can simplify complex information or are more self-explanatory than wordy* | 4.5 |
| *Flowcharts (also called algorithms)* | 4.8 |
| *Graphs enhance interpretation and clarity of the recommendations in an intuitive way* | 4.2 |
| *Tables improve clarity and make documents easier to read* | 4.4 |
| *Use optimized colours and colour coding to draw attention to key words or sections, and in pictorial/graphical displays* | 3.9 |
| *Ensure high contrast* | 3.8 |
| *Use bulleted lists to simplify and clarify a series of points, and to deal with repetition or complex paragraph structures* | 4.7 |
| *Use boxes to display key points* (to improve clarity and usability) | 4.7 |
| *Ensure that there are no awkward breaks of sentences and words in the guideline* | 3.9 |

^a^Action items (individual actionable recommendations with explanatory operational definitions and examples) are italicized

^b^Likert scale: 1 = not at all important; 2 = not important; 3 = neutral; 4 = Important; 5 = extremely important
