# Supplementary material for: The Guideline Language and Format Instrument (GLAFI): development process and international needs assessment survey
Source: Implement Sci. 2022 Jul 19;17:47. doi: 10.1186/s13012-022-01219-2 (PMC9295534; doi:10.1186/s13012-022-01219-2)
Supplement: Supplementary file 3 — Additional file 3. The Guideline Language and Format Instrument (GLAFI). [file 13012_2022_1219_MOESM3_ESM.pdf]

The Guideline Language and Format Instrument (GLAFI)©

Complete sections 1,2,3 for each recommendation and sections 4,5 for guideline as a whole

✓	NA	Action	Operational definition/Example
**1. LANGUAGE: Simple**			
**1a. Succinct and uncomplicated:** The language used in guidelines must be clear, simple, and succinct. Too much information can overwhelm the reader and obscure the final message(s). Complex guidelines may hinder understanding, are less persuasive, and thus more difficult to implement, resulting in lower adherence to recommendations.			
☐	☐	Avoid recommendations requiring many steps, multiple action types, and/or many different conditional factors influencing performance	NA
		**Limit** the number of distinct elements (e.g. conditions) or alternatives provided in a recommendation	Ideally, use **no more than 3 items** or conditions within a recommendation[1-3]; **5 should be the limit.** If **exceeding 5 conditions**, use formatting tools such as **bullets** (see Format section)[4].
		Use **conditional statements** (if, then or if, then, else) to reduce complexity[5]	**For example:** *We recommend that the initial test for ischemic heart disease in patients with LBBB or ventricular paced rhythm should be either vasodilator stress myocardial perfusion imaging or cardiac CT angiography.*<br>Improved Statement: ***If** a patient has a LBBB or ventricular paced rhythm, **then** we recommend vasodilator stress myocardial perfusion imaging or cardiac CT angiography for initial ischemic heart disease testing.*
		Limit any **checklists to 5 to 7 items** to optimize memory[6]	NA
**2. LANGUAGE: Clear**			
**2a. Actionable/Effective writing:** An actionable recommendation is one which provides clear instructions on the action to be undertaken, and the level of certainty with which it is being recommended. It also ensures proper grammar and tone (active voice) to optimize the efficiency of messages.			
☐	☐	Use the **active voice** to make instructions more actionable	**Avoid passive voice:** *A 12-lead ECG should be performed…*<br>**Use active voice:** *Perform a 12-lead ECG…*
☐	☐	If using the **GRADE approach**:<br>Identify recommendations according to their **strength**	Recommendations can be identified as **Strong (Level 1) or Weak (Level 2)**<br>Weak (Level 2) recommendations can alternatively be described using terms such as:<br>- **conditional** (depending on patient values, resources available, and/or setting)<br>- **discretionary** (based on opinion of patient or practitioner)<br>- **qualified** (by an explanation regarding the issues which would lead to different decisions)
		Use an **action verb** corresponding to the strength of a recommendation to operationalize it[7]	For **weak recommendations**, the GRADE working group has suggested less definitive wording, such as "**we suggest**..." or "**clinicians might**..." or "We conditionally recommend…" or "We make a qualified recommendation that…"<br>**For strong recommendations,** the GRADE working group has suggested **terms such as**: *We recommend* or "clinicians should...", "clinicians should not…" or "Do…", "Don't…"
		Employ **consistent use of a letter, number, and/or symbol system** for characterizing both the strength of a recommendation and the quality of evidence	To convey the strength of a recommendation the GRADE working group recommends using either a **number** (e.g. "1" for a strong recommendation) or a **symbol** (e.g. ↑↑ for a strong recommendation).

The Guideline Language and Format Instrument (GLAFI)
Copyright © 2021: Gupta S and Kastner M

			To convey quality of evidence, use either a **letter** (e.g. "A" for high quality) or a **symbol** (e.g. ⊕⊕⊕⊕ for high quality)
		**Specific:** Vagueness occurs when the boundaries of a word's meaning are not well defined; use of specific, concrete statements increases the extent to which information is both understood and remembered.	
❒	❒	**Specify** the target behaviour or action that needs to be performed, by whom, for whom, and under what conditions[8]	Ask yourself if the existing recommendation could be interpreted differently by two different readers, and if so, correct it
		**What** precise action is to be performed (define a measurable, recordable action using action-type verbs (e.g. Prescribe; Perform; Educate; Test; Dispose; Refer/Consult; Monitor; Advocate; Prepare; Diagnose)	**For example, the following statement lacks specificity:** *Serum LDL should be lowered aggressively in older adults at risk for CVD events.*<br>Improved statement**:** *In ambulatory adults aged 60 years of age and older **[For whom]** at high risk for CV events (myocardial infarction or unstable angina) (defined as: [definition of high risk]) **[Condition],** we recommend that family physicians **[Who]** prescribe a statin drug to lower the serum LDL by 50% or more from baseline **[What, in Active voice]***
		**Who** is responsible for completing the action in a given recommendation and **for whom** [i.e. for which exact patients it should (and/or should not) be completed][8]	
		State under what specific **conditions** or circumstances (e.g., when and where) the action is to be performed (if more than one might apply) (e.g. clinical setting, given specific laboratory results, etc.)[9]	
		List **exclusions**: Circumstances where the action should **not** be performed (i.e. specific cases requiring an exception to be made)[10]	
❒	❒	**Avoid "weasel" words** (vague and under-specified words or phrases)	**Avoid (or make sure to define) terms such as**: *Adequate, Sufficient, Moderate, Severe, Frequently, Recurring, Probable, Few*
		For conveying ***temporal*** conditions	**Avoid terms such as**: *Rare, Common*<br>**Use specific numbers, or terms such as**: *Never, Always*
		For conveying ***probabilistic*** statements	**Avoid terms such as**: *Unlikely, Probable*<br>**Use specific numbers, or terms such as:** *Impossible, Certain*
		For conveying ***quantitative*** conditions	**Avoid terms such as:** *Few, Many*<br>**Use specific numbers, or terms such as:** *None, All*
		For conveying ***frequencies, maximum and minimum values*** (ceilings and floors)[8,11]	**For example, avoid:** *"…periodic pre- and post-prandial self-monitoring of blood glucose should be performed"*<br>**Use:** *"…perform pre- and post-prandial self-monitoring of blood glucose with each meal and at bedtime for three consecutive days"*
❒	❒	If ambiguity or vagueness is created deliberately ***(deliberate vagueness)*** (i.e. in areas of insufficient evidence or consensus), provide an explicit statement acknowledging the vagueness and state the reasons[12]	**For example:** *For individuals with type 2 diabetes, if receiving medications not associated with hypoglycemia, infrequent SMBG is appropriate.*<br>Improved Statement**:** *If a patient with type 2 diabetes is not receiving medications associated with hypoglycemia (defined by X), then we recommend once- or twice-weekly SMBG.**<br>*there was insufficient evidence to support a specific SMBG frequency, therefore this frequency was determined by consensus

Copyright © 2021: Gupta S and Kastner M

		**Unambiguous:** Ambiguity can arise when recommendations do not clearly and consistently specify what to do or clarify the parameters on which decisions are based.	
❒	❒	Avoid ***syntactic ambiguity*** (ambiguity caused by the structure of syntax such as lack of punctuation, especially when using Boolean connectors)[13]	**Use Boolean connectors (AND / OR / NOT) along with appropriate punctuation**: *For the statement: "A or B and C", specify if this means (A or B) and C versus A or (B and C)*
❒	❒	Avoid ***pragmatic ambiguity*** (when guidance is not pragmatic because two or more recommendations within a guideline conflict with one another or a recommendation does not include instructions for common clinical scenarios)[13]	**Ensure:** that two or more recommendations within a guideline addressing overlapping actions, target populations, and/or conditions are consistent with one another.<br>**If a specific recommendation or set of recommendations excludes a commonly encountered clinical scenario (i.e., target population and/or conditions):** attempt to provide guidance for the missing clinical scenario (if required, acknowledge the lack of evidence for that scenario and provide consensus guidance)
**2b. Consistent use of Terms**: Ensure that the same terms are used across recommendations whenever possible, and that these terms are used consistently (i.e. to indicate the same meaning).			
❒	❒	Use the same ***semantic indicators*** (use the same terminology to indicate level of evidence, strength of recommendation, and the same action verbs) across recommendations	NA
❒	❒	When comparing alternative approaches, ***frame the recommendations*** in favor of a particular management approach rather than against an alternative	**For example, avoid:** *"In patients with asthma who have suboptimal control (defined as X) on ICS monotherapy, we recommend switching to ICS/LABA therapy, and not doubling the ICS dose."*<br>**Use:** *"…"In patients with asthma who have suboptimal control (defined as X) on ICS monotherapy, we recommend switching to ICS/LABA rather than doubling the ICS dose."*
❒	❒	***Reserve use of "not"*** for recommendations against a management approach that may be particularly harmful and/or widespread	**An example of appropriate use:** "We recommend not initiating inhaled corticosteroids in patients with COPD who have a low symptom burden (mMRC ≤1 and CAT < 10) and are at a low risk for exacerbations (≤ 1 moderate exacerbation and no severe exacerbation in 12 months)."
		**3. LANGUAGE: Persuasive***	
**3a. Framing:** Framing refers to the context in which an issue is presented.			
❒	❒	When justifying a recommendation, place **emphasis on the beneficial outcomes** to be gained from adopting the recommendation (as opposed to the deleterious effects of not adopting it)[14-16]	NA
**3b. Relative advantage:** Behaviour change Is most likely when the reader Is effectively convinced of the advantage of the newly recommended practice over the existing one.			
❒	❒	When a recommendation calls to change a previous, established practice to a new practice, conceptualize the **benefits of the new practice over the previous one in multiple domains** (where possible)[16,17]	**Present the benefits of the new practice in terms of:**<br>• Improved patient outcomes<br>• Economic benefits (from the patient, clinician, and/or system perspective)<br>• Peer acceptance<br>• Ease of use<br>**Provide language:** which recognizes and attempts to mitigate any limitations to adopting the new practice resulting from limited existing resources

*Applies to guideline text other than recommendation alone (e.g. explanatory material around recommendations)

Copyright © 2021: Gupta S and Kastner M

		**4. FORMAT: Components**	
**Standardized components:** Including certain standardized components in guidelines can increase the ease of access for guideline users.			
❒	❒	**Ensure that the following components are included in the guideline**[18-20]**:**<br>• Executive summary of key recommendations – the summary should provide information on where to find more extensive explications in the guideline<br>• A clear display of the logos of all involved organizations[21]<br>• A structured abstract<br>• A glossary of defined terms (particularly when terms are not commonly used or when common usage varies or is imprecise)<br>• An algorithm and/or summary capturing the major recommendations considered essential to the whole guideline[22,23]	
		**5. FORMAT: Presentation**	
**Document layout:** Visual elements and information should be placed and arranged optimally and consistently for ease of interpretation and recall.			
❒	❒	Place **pictorial elements** (e.g. tables/graphs/flowcharts) on the left-hand side of documents, and text on the right for ease of interpretation and recall[24,25]**	NA
**Document structure:** Structure relates to the high-level categorization of the components of a recommendation and how recommendations relate to each other.[26,27]. When guidelines are structured in a sequence that mimics the real patient encounter, using real-world conventions, clinicians assimilate the information better.[28]			
❒	❒	Ensure that the guideline has a **clearly identifiable and optimal structure****[26]	**Headings should:**<br>• Be numbered and named appropriately to convey the message in each section<br>• Use up to a maximum of four levels to break up information.<br><u>Example</u>:<br>1. Cardiovascular Disease<br>A. Etiology<br>i. Behavioural<br>**Ensure:**<br>• Clear chunking (grouping) of information: use sequential arrangement or bundling<br>• Ensure standardized usage of formatting indicators such as type sizes and weights (e.g. bold)<br>• Consider structuring by dividing patients into specific subclasses, if relevant[27]<br>• Group specific recommendations near the summary of key evidence for those recommendations<br>• Consider using bold and/or underline to draw attention to all recommendations, or, if applicable, to a subset of recommendations pertaining to the main PICO question(s) covered by the guideline<br>• Report recommendations in a way that is visible and easy to find (i.e. do not embed recommendations within long paragraphs, and consider grouping recommendations in a summary section)
❒	❒	Present information in an expected and **clinically relevant order** (match the guideline to the real world)	**Use:** a stepwise approach, which presents information in small steps that are matched to the order of actual patient care (from the initial evaluation or presenting complaint to return to function)[28].
		**Grouping/ordering:** Bundling information can reduce effort required to remember a list of recommendations, given limited memory span.[4]	
❒	❒	When a single recommendation or set of recommendations contains many different types of guidance, **bundle similar types of guidance together**	**Example:** If a set of recommendations calls for a total of nine different actions:<br>• Present them in three bundles of three items[4]<br>• Bundle according to type of guidance (e.g. medical tests and procedures in one bundle, pharmacotherapeutic recommendations in another bundle, non-pharmacotherapeutic recommendation in another bundle)

Copyright © 2021: Gupta S and Kastner M

**Information Visualization:** Refers to the visual representation or display of information to enhance usability.[28]			
☐	☐	Replace text explanations with images that can simplify complex information or are more self-explanatory than wordy[24,29]	
☐	☐	**Flowcharts** (also called "algorithms")	**Use flowcharts when decision logic is complex and the temporal sequence of events unclear, to[30]:**<br>• Optimally represent <u>clinical decision pathways</u> or <u>complex information</u><br>• Pictorially describe <u>stepwise recommendations</u> for patient care<br>**Ensure** that any advice that may be seen as ambiguous or counter-intuitive is addressed within the flowchart, through footnotes, or through references to specific sections of the guideline text[5]<br>**Avoid** translating all text information into a flowchart because of its condensed nature
☐	☐	**Graphs** enhance interpretation and clarity of the recommendations in an intuitive way[31]	**When using graphs, choose the best style for the data you are presenting:**<br>• <u>Horizontal</u> bars are best for comparing categorical data (e.g., age group, race, sex, etc.)<br>• <u>Vertical</u> bars are best for comparing ordinal data (e.g., blood group, performance, etc.)<br>• <u>Stacked</u> bar graphs are better at conveying absolute risk<br>• <u>Simple</u> bar graphs are better at conveying relative risk<br>• <u>Line</u> graphs are best when illustrating the effectiveness of a drug or trends over time (e.g., survival/mortality)
☐	☐	**Tables** improve clarity and make documents easier to read)[32,33]**	**Optimized table features:**<br>• Title should be clear and informative<br>• Column headings should be clear<br>• Layout should be consistent and easy to navigate<br>• To facilitate navigation, use bullets to summarize table content where appropriate<br>• Make table format flexible enough to accommodate use:<br>o at the point of care as a quick reference for practice<br>o where relevant, as a tool for shared decision making with patients
☐	☐	Use optimized **colours and colour coding** to draw attention to key words or sections, and in pictorial/graphical displays[34]**	**Use basic colours:** yellow, blue green, red, and black; yellow and blue are best for people who are colour blind<br>**Avoid the colours:** pink, gray, orange, brown or purple<br>**Apply a consistent colour palette throughout the document:** use the same colours and use those colours in the same way (for emphasis, ordering, etc.) in different sections of the guideline
☐	☐	Ensure **high contrast**[34]**	Place a **thin white or black border** around any graphical elements containing coloured shapes in order to enhance contrast<br>**Avoid gray scales,** which are very unreliable as a method of conveying contrast, particularly for quantitative information
☐	☐	Use **bulleted lists** to simplify and clarify a series of points, and to deal with repetition or complex paragraph structures**	**If there are five or more bullets:** consider breaking them into sub-groups of related items[35]<br>**Avoid numbered lists** as this can imply a ranking or preference that may not be intended[35]<br>**Avoid transition words** within bullets.[35] For example:<br>• Firstly,<br>• Secondly,<br>• Etc.…
☐	☐	Use **boxes** to display key points (to improve clarity and usability)[34]**	NA
☐	☐	Ensure that there are no **awkward breaks of sentences and words** in the guideline[32,36,37]**	**Avoid** leaving the first or last line or word of a paragraph on a different page or column

** May require the guideline development group to work with journal editors or editorial staff

Copyright © 2021: Gupta S and Kastner M

References

1. Clark AJ. A Best Practice Guideline for Screening and Treating Vitamin D Deficiency in a Primary Care Setting: Moving Beyond the Bones. https://scholarcommons.sc.edu/etd/323. 2010.
2. Cowan N, Wood NL, Wood PK, Keller TA, Nugent LD, Keller CV. Two separate verbal processing rates contributing to short-term memory span. J Exp Psychol Gen. 1998;127(2):141–160.
3. Luck SJ, Vogel EK. Response from Luck and Vogel. Trends Cogn Sci. 1998;2(3):78-79.
4. Doumont JL. Magical numbers: the seven-plus-or-minus-two myth. IEEE Transactions on Professional Communication. 2002;45(2):123-127.
5. Patel VL, Arocha JF, Diermeier M, Greenes RA, Shortliffe EH. Methods of cognitive analysis to support the design and evaluation of biomedical systems: the case of clinical practice guidelines. J Biomed Inform. 2001;34(1):52-66.
6. Gawande A. The Checklist Manifesto: How to Get Things Right. New York: Metropolis Books; 2010.
7. Brożek JL, Akl EA, Compalati E, Kreis J, Terracciano L, Fiocchi A, et al. Grading quality of evidence and strength of recommendations in clinical practice guidelines part 3 of 3. The GRADE approach to developing recommendations. Allergy. 2011;66(5):588-95.
8. Gurses AP, Marsteller JA, Ozok AA, Xiao Y, Owens S, Pronovost PJ. Using an interdisciplinary approach to identify factors that affect clinicians' complicance with evidence-based guidelines. Crit Care Med. 2010;38(8 Suppl):S282-S91.
9. Shiffman RN, Shekelle P, Overhage JM, Slutsky J, Grimshaw J, Deshpande AM. Standardized reporting of clinical practice guidelines: a proposal from the Conference on Guideline Standardization. Ann Intern Med. 2003;139(6):493-498.
10. Gurses AP, Seidl KL, Vaidya V, Bochicchio G, Harris AD, Hebden J, et al. Systems ambiguity and guideline compliance: a qualitative study of how intensive care units follow evidence-based guidelines to reduce healthcare-associated infections. Qual Saf Health Care. 2008;17:351-359.
11. Rosenfeld RM, Shiffman RN. Clinical practice guidelines: a manual for developing evidence-based guidelines to facilitate performance measurement and quality improvement. Otolaryngol Head Neck Surg. 2006;135:S1-S28.
12. Codish S, Shiffman RN. A Model of Ambiguity and Vagueness in Clinical Practice Guideline Recommendations. AMIA Annu Symp Proc. 2005;2005:146-50.
13. Grapentine TH, Weaver DA. What Really Affects Behavior? Marketing Research. 2009;Winter:11-7.
14. Greenhalgh T, Robert G, Macfarlane F, Bate P, Kyriakidou O. Diffusion of innovations in service organizations: systematic review and recommendations. Milbank Q. 2004;82(4):581-629.
15. Scott SD, Plotnikoff RC, Karunamuni N, Bize R, Rodgers W. Factors influencing the adoption of an innovation: an examination of the uptake of the Canadian Heart Health Kit (HHK). Implement Sci. 2008;3:41.
16. Tornatzky LG, Klein KJ. Innovation Characteristics and Innovation Adoption-Implementation: A Meta-Analysis of Findings. IEEE Transactions on Engineering Management. 1982;EM-29(1):28-43.
17. Shaneyfelt TM, Centor RM. Reassessment of Clinical Practice Guidelines: Go Gently Into That Good Night. JAMA. 2009;301(8):868-9
18. Brouwers MC, Graham ID, Hanna SE, Cameron DA, Browman GP. Clinicians' assessments of practice guidelines in oncology: the CAPGO survey. Int J Technol Assess Health Care. 2004;20(4):421-6.
19. Stone TT, Schweikhart SB, Mantese A, Sonnad SS. Guideline attribute and implementation preferences among physicians in multiple health systems. Qual Manag Health Care. 2005;14(3):177-87.
20. McKinlay E, McLeod D, Dowell A, Marshall C. Clinical practice guidelines' development and use in New Zealand: an evolving process. The New Zealand Medical Journal. 2004;117(1199):U999.
21. Vlayen J, Aertgeerts B, Hannes K, Sermeus W, Ramaekers D. A systematic review of appraisal tools for clinical practice guidelines: multiple similarities and one common deficit. Int J Qual Health Care. 2005;17(3):235-42.
22. Maviglia SM, Zielstorff RD, Paterno M, Teich JM, Bates DW, Kuperman GJ. Automating complex guidelines for chronic disease: lessons learned. J Am Med Inform Assoc. 2003;10(2):154-65.
23. Tu SW, Campbell J, Musen MA. The Structure of Guideline Recommendations: A Synthesis. AMIA Annual Symposium Proceedings. 2003;2003:679-83.
24. Silayoi P, Speece M. The importance of packaging attributes: a conjoint analysis approach. European Journal of Marketing. 2007;41(11-12):1495-1517.
25. Ariely D. Predictably Irrational: The Hidden Forces That Shape Our Decisions. New York: Harper Perennial; 2009.
26. Grimshaw JM, Russell IT. Effect of clinical guidelines on medical practice: a systematic review of rigorous evaluations. Obstet Gynecol Surv. 1994;49(7):469.
27. Harris JS, Meuller KL, Low P, Phelan J, Ossler C, Koziol-McLain J, et al. Beliefs about and use of occupational medicine practice guidelines by case managers and insurance adjusters. J Occup Environ Med. 2000;42:370-6.
28. Kushniruk AW, Patel VL. Cognitive and usability engineering methods for the evaluation of clinical information systems. J Biomed Inform. 2004;37(1):56-76.
29. Quaglini S, Ciccarese P. Models for guideline representation. Neurol Sci. 2006;27 Suppl 3:S240-4.
30. Rosenfeld RM, Shiffman RN. Clinical practice guideline development manual: a quality driven approach for translating evidence into action. Otolaryngol Head Neck Surg. 2009;140(6 Suppl 1):S1-S43.
31. Reyna VF. A theory of medical decision making and health: fuzzy trace theory. Medical Decision Making. 2008;28(6):850-6.
32. National Institute for Health and Clinical Excellence. The guidelines manual. London: National Institute for Health and Clinical Excellence; 2009.
33. Wolff M, Bower DJ, Marbella AM, Casanova JE. US family physicians' experiences with practice guidelines. Fam Med. 1998;30(2):117-21.
34. Ware C. Information Visualization. San Francisco: Morgan Kaufman; 2004.
35. Gaertner-Johnston L. Business Writing With Heart: How to Build Great Work Relationships One Message at a Time. Washington: Syntax Training; 2013.
36. Frascara J, Ruecker S. Medical communications and information design. Information Design Journal. 2007;15(1):44-63.
37. Neville C. Effective Notemaking. Bradford: University of Bradford School of Management; 2006.

Copyright © 2021: Gupta S and Kastner M
